# Transitions: Living With Young‐Onset Alzheimer's Disease: A Qualitative Interview Study

**DOI:** 10.1111/hex.70034

**Published:** 2024-09-16

**Authors:** Malin Aspö, Leonie N. C. Visser, Miia Kivipelto, Anne‐Marie Boström, Berit Seiger Cronfalk

**Affiliations:** ^1^ Department of Neurobiology, Care Sciences and Society, Division of Clinical Geriatrics Karolinska Institutet Stockholm Sweden; ^2^ Theme Inflammation and Aging Karolinska University Hospital Stockholm Sweden; ^3^ Department of Medical Psychology, Amsterdam University Medical Center University of Amsterdam, Amsterdam Public Health Amsterdam The Netherlands; ^4^ Department of Research and Development Stockholms Sjukhem Foundation Stockholm Sweden; ^5^ Institute of Public Health and Clinical Nutrition University of Eastern Finland Kuopio Finland; ^6^ Department of Neurobiology, Care Sciences and Society, Division of Nursing Karolinska Institutet Stockholm Sweden

**Keywords:** Alzheimer, experiences, Meleis, qualitative, transitions, young‐onset dementia

## Abstract

**Introduction:**

Persons with young‐onset dementia (YOD) are confronted with specific challenges. Due to the neurodegenerative nature of the disease, people diagnosed with YOD face many changes with different consequences, for example, regarding their life perspective. These changes can give rise to transition processes and strategies for coping, hopefully stimulating well‐being and acceptance. However, this might not always be the case, and support may be warranted. Our aim was to describe the experiences of those living with YOD due to Alzheimer's disease (AD) and identify signs of transitions during the first year after diagnosis.

**Method:**

In this qualitative interview study, we explore the experiences of younger persons living with AD. Thirteen participants under the age of 65 years (nine female and four male; mean age: 57) were included 1 year after being diagnosed with AD. The interviews were transcribed verbatim and analyzed using qualitative content analysis with a deductive approach. To gain a deeper understanding of the data, Meleis's transitions theory was used as a theoretical framework.

**Results:**

Two categories were identified: ‘Life has changed’ and ‘Mastering a changed life situation’. One year after diagnosis, participants described how they experienced a changed life situation, changing symptoms, a loss of meaningful activities and an increased risk of social isolation. Furthermore, living with uncertainty about the future caused feelings of being disconnected. Awareness was described as an important aspect of coping with YOD and progressing in the transition process. Participants also highlighted the importance of support from others.

**Conclusion:**

The results suggest that preventing social isolation is important in facilitating healthy transitions. Therefore, professionals need to identify signs of transitions and be aware of the complexity of coping with YOD, thereby helping to prevent unwanted responses to change and facilitate a healthy transition process.

**Patient or Public Contribution:**

The findings are based on interviews with 13 persons with YOD and provide insight into experiences of living with YOD.

**Abbreviations**
AD: Alzheimer's disease
MMSE: Mini‐Mental State Examination
MoCA: Montreal Cognitive Assessment
YOD: young‐onset dementia

## 1 Introduction

Receiving a diagnosis of dementia is described as the starting point of a transition process towards adapting to a changed life perspective. The most common cause of dementia is Alzheimer's disease (AD) [1]. Of those living with AD, 5%–6% were diagnosed before the age of 65 years [2], thus, living with young‐onset dementia (YOD). In total, YOD accounts for up to 9% of all dementia cases [3]. As the disease progresses, several critical stages are likely to occur. Some are possible to predict as they are directly linked to disease progression and related to decisions regarding future needs and issues, such as driving license, behavioural changes and changes in care settings [4]. However, the disease trajectory is different for every person with YOD, and individuals might also experience disease‐related changes differently.

Dementia has a great impact on a person's life and the lives of relatives and friends, and this impact can be even greater if the person is diagnosed with YOD [5]. One explanation may be that younger persons are more likely to be part of the workforce and have financial responsibilities, such as taking care of a family, possibly with young dependent children [6, 7]. One dominant critical point is the forced withdrawal from professional life [8, 9, 10] because this may have financial consequences [8, 11] and cause the loss of meaningful activities that are often difficult to replace [12]. In addition, persons with YOD experience challenges in identifying with the stereotype of a person with dementia [13, 14]. Despite the specific challenges and needs of persons with YOD, there is a lack of specific support, such as services that facilitate social engagement or address legal or financial aspects [15, 16]. The need for post‐diagnostic support is especially highlighted by Bakker et al. [17].

In previous research [12, 18, 19], maintaining continuity and a sense of ‘normal’ life after being diagnosed with dementia have been considered important aspects of coping in the early phases of YOD, for example, by staying engaged and feeling useful [20]. However, as the disease progresses, continuing previous roles and tasks becomes too demanding [19], and maintaining these is no longer a sufficient coping strategy. In previous research, coping with dementia has mainly been described from the caregiver's perspective [18, 21]. Therefore, we know little about how persons with YOD cope with the various changes they are confronted with as dementia progresses and symptoms worsen.

Meleis's [22] transitions theory helps gain a better understanding of how persons with YOD adapt to their altered life situations. The theory describes a transition as a disruptive period between two stable periods or the passage from one phase of life to another. Meleis [22] has defined five properties that are signs of an ongoing transition process: ‘change and difference’, ‘time span’, ‘critical points and events’, ‘awareness’ and ‘engagement’. All transitions take place over time and are initiated by a critical point at which the person first shows signs of awareness and engagement in the transition process. During the transition, the person experiences a state of disconnectedness and vulnerability, as well as being confronted with the different aspects of feeling changed as a person [22]. This may include alterations in the identity, roles, abilities, relationships or health of the person in transition. Indicators of a healthy transition are emotional well‐being, mastery of new roles and skills and good relationships. Patterns of healthy responses to a transition are described as feeling connected, interacting, being situated and developing confidence and coping. The main focus of nursing is to support health and well‐being, such as by facilitating healthy transitions [22].

To provide appropriate support to younger persons with dementia, it is important that professionals have knowledge of the specific needs of YOD. Because AD is a progressive disorder in which several critical points are to be expected, professionals who meet persons with YOD, both within and outside the healthcare sector, require knowledge and understanding about potential transitions. In addition, professionals need to recognize a person's ability and readiness for an upcoming transition and explain what to expect from the process. During the transition process, it is also important to identify whether the person is moving towards a healthy transition or increased vulnerability [4, 22]. Therefore, the aim of this study was to describe the experiences of persons living with YOD due to AD and to explore signs of transitions during the first year after receiving the diagnosis.

## 2 Methodology

### 2.1 Study Design

This study used a qualitative design with semi‐structured interviews focusing on the experiences of younger persons living with AD. The study is part of a 5‐year longitudinal project collecting data on experiences and quality of life in a sample of persons with YOD. Participants were included at the time of receiving their diagnosis to participate in yearly interviews during the project. The results of the present study are based on data from the second interviews that were conducted 1 year after diagnosis.

### 2.2 Setting

Participants were recruited from two specialized memory clinics at a university hospital in Sweden. Persons are referred to these clinics following a basic evaluation by their general practitioners. At the specialized clinics, patients undergo a standardized cognitive evaluation, including cognitive testing, medical examinations, assessment of biomarkers and MRI. Approximately 25% of the patients are diagnosed with dementia [23].

### 2.3 Participants

Initially, 15 participants with YOD were recruited and interviewed shortly after being diagnosed. Inclusion was based on the following criteria: persons ≤ 65 years of age, recently diagnosed (≤ 2 months) with dementia with no previously diagnosed condition of impaired cognition; a MMSE score of ≥ 24; ability to communicate verbally and in writing and ability to provide verbal and written consent. One year after diagnosis, one participant had withdrawn, and one could not be reached. In total, 13 participants diagnosed with AD participated in the interviews for this study.

### 2.4 Data Collection

Interviews were conducted between June 2019 and September 2021 by the first author, who had also conducted the initial interview with the participants. Before the current interviews, the first author re‐read the first interviews thoroughly and prepared questions that would follow up on critical points identified in the first interviews. In addition, all participants were asked questions regarding their current life situation and their experiences since the previous interview. The interviews were audio recorded and transcribed by the first author and a research assistant. Due to the COVID‐19 pandemic, after March 2020, most interviews were conducted by phone.

### 2.5 Analysis

A qualitative content analysis with a deductive approach was performed [24, 25]. The following categories were identified in the first interviews [8]: ‘A life changing moment’, ‘An ongoing process’ and ‘Remaining in control’, used as an initial framework for coding the data. Following the steps presented by Granheim, Lindgren, and Lundman [24], the transcripts were read several times to gain a deeper understanding before identifying units of meaning. These were then condensed, coded and grouped into sub‐categories and categories, inspired by categories identified in the first interviews [8]. These categories were then revised to better reflect the changes participants described to have occurred during the year that had passed since receiving their dementia diagnosis. Meleis's [22] transitions theory was used as a theoretical framework to identify signs of transitions. The focus was on describing and interpreting the findings through the lens of the properties of the transition experience: ‘change and difference’, ‘time span’, ‘critical points and events’, ‘awareness’ and ‘engagement’.

The analysis was conducted in close collaboration between the first and last authors, who coded the interviews independently and had regular meetings to discuss the process, before reaching consensus regarding sub‐categories and categories. Co‐authors have been involved in the process by critically reviewing the results. All authors have extensive experience of meeting persons with dementia. The authors reflected on their pre‐understanding and evaluated if their knowledge in any way influenced the coding and interpretation of the findings. In this way, the reported sub‐categories and categories truly reflect the experiences of the participants rather than the aspects of the authors' prior knowledge.

### 2.6 Ethical Considerations

The study was approved by the Ethics Review Board in Stockholm, Sweden (Dnr: 2017/2400‐31/1) and conducted in compliance with *The Declaration of Helsinki* [26]. Participants provided written informed consent once, before the start of the longitudinal study, after being informed that this would involve interviews at multiple time points. At the start of the current interview, the first author again informed that participation was voluntary and confirmed that the participant agreed to participate.

## 3 Results

In total, 13 persons (nine women and four men) with an average age of 57 years, all diagnosed with YOD due to AD, participated in the current interviews. Table 1 presents the sample characteristics. At the time of the interview, 1 year after their dementia diagnosis, all participants were still living in their private homes, and most were still experiencing mild symptoms. For most participants, data from MMSE and/or Montreal Cognitive Assessment (MoCA) were only available at the time of diagnosis.

**Table 1:** Sample characteristics.

Sample characteristics	
Sex	
Female	9
Male	4
Age mean (range)	57 (50–66)
MMSE mean (range)	
At diagnosis (*n *= 12)	27 (24–30)
A year after diagnosis (*n *= 5)	23 (21–26)
MoCA mean (range)	
At diagnosis (*n *= 12)	23 (15–26)
A year after diagnosis (*n *= 7)	23 (17–27)
Employment status (*n*)	
Employed full time	1
Employed part time	2
Unemployed	1
On sick leave	5
Early retirement	4
Marital status (*n*)	
Married	9
In a relationship, not living together	1
Widowed	1
Single	2
Children (*n*)	
Having children younger than 18	2
Having children older than 18	11

The findings in this study focus on experiences and signs of transitions and will be presented in two categories: ‘Life has changed’ and ‘Mastering a changed life situation’, each with encompassed sub‐categories (Table 2).

**Table 2:** Categories and sub‐categories.

Categories	Sub‐categories
Life has changed	Changes in symptoms
	Loss of activities
	Living with uncertainty
Mastering a changed life situation	Reduced focus on the disease
	Coping with change
	Understanding and support from others

### 3.1 Life Has Changed

In this category, participants described how living with YOD had altered different aspects of their lives during the year since diagnosis. These changes were described as related to changes in symptoms, loss of activities and uncertainty due to the inability to control the progression of the disease.

### 3.2 Changes in Symptoms

Changed symptoms were described as a main factor for how the participants experienced the gradual progress of their disease. Impaired short‐term memory was described as the most prominent symptom, followed by symptoms related to language and apraxia, because this affected everyday life. The participants were more vigilant and aware of new symptoms being signs of a disease in progress. The loss of functions was also described as a constant reminder of the disease, causing feelings of anxiety.

It is that one must live with this, the anxiety, and somehow you notice the changes. And that is depressing every time I notice that ‘oh no, I'm getting worse now’. You become aware of the decline.

(Participant 6)

As the participants' awareness increased, so did their conscious efforts to prevent the worsening of symptoms. They described how the severity of symptoms often increased when they felt stressed. Taking care of basic needs, such as sleep and avoiding stressful situations, were therefore described as important. One participant reported improved cognitive functions and reflected on how this might be a result of life now being less stressful.

I was there (at the memory clinic) in February and did some tests and my score had improved since the time before. And maybe that was because I'm calmer now because the sick benefit is organized. I feel that it's given me peace of mind and I'm not as tired as before.

(Participant 2)

Avoiding stressful situations, such as travelling by public transport due to the fear of getting lost and not understanding timetables, was described as impacting the participants' social life. As these difficulties increased, participants described that they withdrew from social activities outside their local environment. Some were no longer allowed to drive and described how this affected their engagement in social activities.

I wouldn't go to town on my own, I wouldn't. Because I wouldn't find the way. I would not find my way at the subway station as it has changed and is not as it used to be.

(Participant 1)

### 3.3 Loss of Activities

Due to the cognitive impairments, most participants were no longer working, and this was described as having had a significant impact on everyday life. The paradox was that, although not being exposed to a stressful environment faced with difficulties was described as a relief, it still generated feelings of loss. However, some expressed having accepted that their professional working life had come to an end.

I've been working all my life and I've loved it […] But then life takes a different turn and I've no problem with that now. No no, that (the anxiety) was at the beginning.

(Participant 5)

Those who had stopped working experienced difficulties replacing work‐related activity with other meaningful activities. Because most family members and friends were away from home working during the day, participants spent most of the daytime hours by themselves. They described how this increased their feelings of distress and worries about becoming isolated.

It's my choice; I own half the company, so I'm okay. I manage financially and so do they. But what the hell shall I do all day long? Sometimes half days but ‘what the heck is this?’, ‘what do I do?’, ‘this is simple stuff to do but why can't I?’ It's really hard, really hard.

(Participant 6)

For those still working, it had become evident that, over time, their cognitive symptoms affected them, especially in stressful situations. One described the need to focus harder than before, whereas another had reached a point where it was no longer possible to continue working. One participant, who had recently stopped working, described the abrupt end to their working life as difficult to accept. The participant described the process of leaving their working life and being on sick leave as too quickly, not allowing time for reflection about the consequences.

It's an important decision to make, do you take sick leave or carry on working. Wow, what shall I do? That's how I felt. Perhaps more time to reflect together would have been good. Instead, it was ‘you're on sick leave’. What does that mean? […] It was like cutting off my hand straight away.

(Participant 9)

The financial consequences of no longer working were especially profound for those living on their own. Participants described various difficulties receiving the financial support they were entitled to, for example, sickness benefits. Some described that they had to spend their life savings to survive. The participants who had been on sick leave before their diagnosis, and who had now entered early retirement, described a sense of relief at no longer having to deal with the administrative complications of receiving financial support from the Swedish Social Insurance Agency or problems with their employers.

It was a relief to get that sickness benefit, because then I didn't have to keep arguing with the Social Insurance Agency or with the Employment Service or anything like that. So, then you could put it (those worries) aside.

(Participant 2)

### 3.4 Living With Uncertainty

When being diagnosed with AD, participants confronted their own perceptions of dementia and how they would be viewed by society and described having an altered view of themselves. This was especially profound when describing personal relationships, such as the disease putting an end to the possibility for those who were single of meeting a partner. Several participants also described their frustration with how society often treats persons with AD as old and fragile. The participants did not want to be associated with these stereotypes.

I have no desire to be treated like an 80‐year‐old. Because that's what I'm experiencing, I was demoted in two seconds. From a lively and happy 58‐year‐old to something very close to death.

(Participant 14)

Receiving the diagnosis inevitably forced the participants to realize that their lives would not turn out as previously expected. They experienced feelings of uncertainty regarding the disease progression and wished to receive more information about the expected trajectory. However, although learning more about the disease in the beginning was seen as a way of remaining in control, some participants now described that new information could be a source of anxiety.

It was after I had read that book (Still Alice), where there was a very accelerated progression, that I became worried. Then I thought ‘Oh, will I be like a vegetable in two and a half years?’ I'd never realized that. In my mind, it's always been like 7‐8 years in the future before I might need care.

(Participant 13)

Most participants experienced a slow progression of their disease and described having learned to manage their symptoms in everyday life. Yet, the fear of a rapid decline in functioning made participants reflect on the later phases of dementia. Becoming dependent on others and not being able to communicate one's own needs caused a lot of distress. A few participants even mentioned euthanasia as a possible alternative to avoid the later phases of dementia.

So eventually I was actually so sad that I made the decision that I'm going to stay alive until the day I think it's no longer worthy. And I've told my kids and my best friend […] there will come a day when I understand that, now I can't live a normal life anymore and, and I'm not prepared to go into a life other than the one I had.

(Participant 14)

In summary, the findings highlight various changes that participants had experienced in their life during the year after being diagnosed with YOD. Being compelled to withdraw from working life and their voluntary withdrawal from social activities were factors that increased the risk of social isolation. Furthermore, living with uncertainty about the future and the inability to control it caused feelings of anxiety and disconnection.

## 4 Mastering a Changed Life Situation

The participants described using different strategies to cope with and master their changed life situation. Accepting the diagnosis was described as a process that needs time in which awareness was described as a crucial component. However, the level of awareness and acceptance varied among the participants. Some also described needing support from others to master their changed life situation.

### 4.1 Reduced Focus on the Disease

Immediately after receiving their dementia diagnosis, thoughts about the diagnosis and its consequences were constantly on the participants' minds. These thoughts were no longer as intense, and participants described the passing of time as important in the process of accepting the diagnosis and integrating it as part of their altered life situation.

It's been a process this year and it's gone through the usual stages, so to speak, after you get a message that's shocking […] then came the period when you had to process it and tell those closest, and would you tell or not tell […] and then there was a period of non‐acceptance like, ‘no, this can't be right’, it was hard to take in.

(Participant 13)

Overall, the need for information about the disease was described as having decreased. Participants who were still actively searching for information described the same purpose for doing this after receiving the diagnosis, for example, to learn more about dementia and what they could do to slow down the progression. Participants also described a more relaxed approach towards the decision of sharing or not sharing information about their diagnosis with others. Most had already told others, whereas some expressed not being in a hurry to disclose the diagnosis until their symptoms increased.

I think people look at you with slight disbelief actually, if you say you have Alzheimer's, so that's something I should keep to myself as long as possible I think.

(Participant 2)

Participants described awareness of the disease as crucial in their process towards acceptance. However, some showed signs of lacking disease awareness or of not wanting to accept the cause of their symptoms and expressed that they were looking for alternative explanations. Acknowledging the disease as the cause of everyday difficulties was also described as an important factor in the process of accepting the need for extended support.

I didn't want to go to the day care center then because it felt a bit too early in some way, or what shall I say, ‘there's nothing wrong with me’ […] it's like you're in kindergarten, but now I would probably think it would be really nice to be there. To meet others in the same situation and you can talk and laugh and, yes, have a nice time as well. It would mean a lot.

(Participant 4)

### 4.2 Coping With Change

Participants described using different strategies for controlling and coping with their changed life situation. Since receiving the diagnosis, one participant had joined a support group and described how this resulted in improved feelings of social engagement. Everyday coping strategies described mainly included shifting responsibilities within the family, engaging in activities that distracted them from negative thoughts, focusing on positive aspects in life and seizing the day.

I've noticed that it's the best way, to be social and do stuff. And then when you come home and you're tired then you go to bed and sleep. Like that, you don't have to think about it. So that it's a way of doing something, of being busy, then you don't think about it so much.

(Participant 6)

Additional strategies described were related to preparing for the future, such as arranging for more convenient living arrangements, financial planning and signing a will. The possibility of signing a lasting power of attorney was also mentioned, including the uncertainty surrounding when and how this would take effect.

I just thought it was so incredibly vague about when it would come into force, because after all, I'm the one who decides when it will take effect. And it's also so damn fuzzy if you have Alzheimer's! Are you demented or are you not?

(Participant 2)

The importance of not giving up on the hope of being able to affect disease progression was also expressed. The participants described putting faith in a recent approval of drug treatment in the United States. They also described making conscious decisions regarding their lifestyle, even if not as strict now as immediately after receiving the diagnosis. Some reported noticing positive effects of their healthier lifestyle on their physical health and cognition.

It is much easier to focus […] I feel a lot more, how should I put it, energy. I can cope better. I'm more awake.

(Participant 3)

Most participants described having strategies to manage symptoms and emotions and cope with daily life. However, one participant clearly expressed not being able to cope without support from others, as being alone during the daytime caused anxiety. The participant wanted to attend a specialized day care centre to lessen the burden on their family; however, this was neither acknowledged nor approved by the local care administration.

If I was able to go to a day care center twice a week, at least they wouldn't have to worry about me not eating or not taking my pills, or if I've closed and locked the door after me.

(Participant 4)

### 4.3 Understanding and Support From Others

The participants described their families as important and a source of support. Some had become more dependent on support from the family in daily life. This was mainly through family members reminding them about things they had forgotten, as well as supervising daily routines. Receiving the dementia diagnosis provided an explanation of the participant's symptoms and behaviour, which positively influenced the family members’ understanding in most cases.

It used to be how we communicated with each other. I could be really angry with him. I would say ‘I just can't remember’ and then we argued, but as soon as he knew he's been fantastic, so helpful and caring. So, it works really well, and we very seldom have any arguments.

(Participant 3)

However, participants also addressed challenges in receiving support and understanding from family members. This included not being able to talk about emotional and existential issues or avoiding sensitive subjects, such as their fear of having to move to a residential care home in the future, as a means of not increasing the emotional burden on their family members. Some participants described conflicts within their families due to a lack of understanding; for example, one participant's sister distanced herself, the aged parents of another could not understand or respect boundaries and a participant was no longer allowed to spend time alone with their grandchildren, which caused great sorrow.

It was a shock to my children initially, that's understandable but to hear ‘just so you know you'll never be allowed to be alone with them (grandchildren)’. I went home and cried.

(Participant 5)

Participants described being satisfied with the current support they received from the memory clinics. They found it easy to get in contact with the clinic, if they needed to, outside of their routine check‐ups. Nevertheless, several highlighted that the support given in the initial period after receiving the diagnosis needs to be improved, as most had left the memory clinic with several unanswered questions. Some participants suggested that the clinics should arrange lectures to provide patients and their families with reliable up‐to‐date information.

Yes, I needed a lot (information after diagnosis). Because that information didn't come automatically. I believe everyone would have needed to get it. When you're not that old either. What it will look like, roughly, it's not possible to say exactly. But it was just ‘smack’ ‘you've got Alzheimer's, goodbye!’

(Participant 5)

In summary, this second category describes the acceptance of the diagnosis as a process occurring over time and draws attention to the importance of participants' awareness of the disease process. This category also highlights the complexity of coping with dementia and the importance of support and engagement from others (family and professionals).

## 5 Discussion

Being diagnosed with YOD was described as a critical point that has a drastic impact on participants' life. Our findings provide an insight into the altered life situation, transitions and critical points during the first year after the diagnosis. The findings highlight the challenges of mastering a changed life situation after being diagnosed with YOD, due to the ever‐changing nature of the disease, as well as how the disease itself impacts the person's ability to adopt adequate coping strategies. The participants showed signs of healthy transition processes and signs that might have negative impacts, such as the tendency to isolate socially. To facilitate healthy transitions, it is important that professionals can identify signs of transitions and are aware of the complexity of coping with YOD.

Different strategies were described for coping with their changed life situation. The mastery and development of new skills became evident as the participants showed improved awareness of their limitations and engaged in developing strategies to control their new life situation. The participants also described different strategies for prolonging the initial, mild phase of AD dementia by, for example, searching for information about how to prevent further cognitive decline, resulting in a healthier lifestyle for some. Showing engagement in the process and adopting coping strategies is according to Meleis [22], a sign that the person is moving towards a healthy response to the transition.

Meleis [22] describes the importance of being situated, such as creating meaning and understanding in the new situation, as an important aspect of a healthy transition. After receiving the diagnosis, participants described how thoughts about dementia occupied their minds constantly [8]. Our current results suggest that the reduced focus on the disease may be a sign of acceptance and participants not letting YOD define them as a person. It may also be a sign of them shifting their focus towards trying to maintain continuity and a sense of normality. This has been addressed by Johannessen et al. [19] as an important means of preserving one's identity. However, considering the complexity of coping with dementia, it may also be a sign of avoidance and denial [18]. Avoidance may be experienced as beneficial because it reduces the emotional burden; however, avoidance may also hinder the person from proceeding in the transition process through not accepting their altered life situation and not dealing with the consequences optimally [22].

Due to the complexity of coping with dementia, strategies that the participants consider as beneficial at that moment may have negative long‐term effects [27]. The most prominent example was the avoidance of stressful situations as a means of reducing the cognitive burden in daily life. This, along with the premature end to working life, consequently led to participants withdrawing from social activities, which increased the risk of isolation and vulnerability. Avoiding stressful situations and the increased risk of isolation as the disease progresses is well known [12, 18, 19], and Johannessen et al. [19] have identified forced withdrawal from working life as a starting point of social retraction. In the first interviews, the participants had a greater focus on the financial consequences of the withdrawal from working life [8], whereas in the present interviews, the focus was more on the loss of meaningful activities. This is in line with the conclusions drawn by Greenwood and Smith [12] regarding the importance of replacing working life with other meaningful activities. People might need guidance to help them replace activities and stay engaged in social activities, particularly when others around them are occupied during the day, which is especially relevant to those with YOD.

Professionals need to be able to identify signs of unwanted responses to the transition process, such as avoidance and the tendency for social isolation. Greenwood and Smith [12] conclude that persons with YOD have a strong wish to stay engaged in meaningful and social activities, and Johannessen et al. [19] highlight the importance of continuity. However, this may become challenging as the disease progresses, and tasks/roles previously held become too demanding [19], leading to further withdrawal and isolation. In our study, participants described a wish to prolong the initial phase of the disease. Research has shown that engaging in social activities may reduce the risk of cognitive decline and dementia [28], making the prevention of isolation even more important.

Meleis [22] addresses the importance of feeling connected as a sign of a healthy transition. This includes making new contacts and maintaining established connections with family and friends. This is in line with the findings of Mayerhofer et al. [15], which state that support and services should focus on social inclusion and creating a sense of ‘normality’. Work‐based support programmes [29, 30] may be one way of facilitating a sense of 'normal life’ and creating a sense of purpose for persons with YOD who are no longer able to work. In addition, the support and education of employers may increase the possibility for persons in the early stages of AD to remain in the workforce, for example by adjusting tasks to the person's needs and abilities [31]. Feeling connected to family and friends is also important as a means of facilitating continuity and connectedness. In our study, most family members were described as being understanding and supportive; however, a few participants described conflicts with family members due to their diagnosis. Johannessen et al. [19] found that social relationships could often be experienced as complicated and destructive and, therefore, ended by the person with dementia, leading to increased social isolation and less identity support.

Healthcare professionals could also help persons with YOD to feel connected by answering questions and providing support [22]. We have previously [8] suggested that the work of memory clinics should focus on facilitating successful transitions. Considering the findings from the present study, we suggest that during the first year after receiving a diagnosis of YOD due to AD, support should be introduced using a step‐wise approach. Feelings of abandonment that were experienced immediately after being diagnosed were no longer an issue after a year, so the immediate support should be focused on reducing these feelings and creating a sense of security and reassurance. This includes providing the persons with both reliable information about AD and support with handling practical and financial issues [8]. This suggests a need for more frequent contact with the memory clinics at the beginning of the transition.

The participants described that the support they received from the memory clinic after a year met their expectations. This indicates that the frequency of contact might be phased out as the person develops adaptive coping strategies. However, we identified the risk of social isolation as being an important indicator of an unwanted response to the transition process. We believe that several factors associated with the risk of social isolation could be avoided by facilitating engagement in social activities and supporting everyday roles and identity, including preserving personal relations. This could, for example, be accomplished by supporting adaptive coping strategies and ensuring that persons with YOD have access to services and meaningful daytime activities. We would suggest that this should be prioritized as an area of interest by professionals meeting persons with YOD.

It is important to note that support needs to be personalized and adapted to the individual's specific needs, taking psychosocial aspects and the person's cognitive function into account. Participants in our study were in different phases of the disease when they received their dementia diagnosis. There was also variation in how participants coped with changes caused by the disease. Our findings show that already during the first year after diagnosis, persons may lack effective coping strategies and need support in coping, in line with the findings of Johannessen et al. [19]. This highlights the need for personalized support to facilitate transitions by supporting the persons in mastering their altered life situation. This support should be provided by a professional, with knowledge of the complexity of coping with YOD, who can guide the person through the disease trajectory by coordinating care and access to services and creating a sense of security [32, 33].

### 5.1 Strengths and Limitations

The main strength of this qualitative study is its longitudinal design. The study contributes to the area of research concerning persons' experiences of living with YOD and provides an opportunity to study how needs change over time as dementia progresses. Because all participants had been interviewed shortly after receiving their diagnosis [8], we were able to describe and compare experiences with the first interviews. This increases the possibility of capturing transitions compared to if the interviews had only been conducted 1 year after diagnosis. There are some limitations that can affect the transferability of our findings. First, only persons with AD were included in the study. Compared to late‐onset dementia, the prevalence of other types of dementia is higher in YOD [34], and our findings may not represent the experiences of persons with a different type of dementia. Second, most participants in the study were living with a partner and had adult children; persons living without a partner or with young children may experience other challenges not identified in our interviews. Due to the COVID‐19 pandemic, several interviews had to be conducted by phone. However, we believe that this change had limited impact because a relationship between the first author and the participants had already been established during the first interviews.

## 6 Conclusion

As dementia progresses, the person will experience different types of transitions as their symptoms and needs change. Professionals in contact with persons with YOD could facilitate healthy transitions, by preparing them for future critical points, detecting early signs of change and transitions and supporting the person in ongoing transitions. A year after diagnosis, participants described no longer feeling abandoned by the memory clinic staff and a reduced need for dementia‐related information. We conclude that the immediate post‐diagnostic support should focus on enhancing the person's knowledge of the disease as a way of regaining control and mastery through more frequent contact with the clinic. Furthermore, after the initial period, our current findings suggest that support should focus on preventing social isolation and providing the person with meaningful activities. More research is needed to investigate how social isolation can be prevented and what type of meaningful activities persons with YOD could benefit from. Lastly, there is a need for increasing awareness and knowledge of YOD by persons in authorities outside the healthcare sector and for developing clear guidelines for professionals within the social services sector that account for the neurodegenerative nature of AD. This further highlights the need for more longitudinal research because this provides a unique opportunity to gain an in‐depth understanding of how persons with YOD experience transitions in different phases of dementia.

## Author Contributions

**Malin Aspö:** writing–original draft, conceptualization, investigation, formal analysis, project administration, data curation. **Leonie N.C. Visser:** conceptualization, formal analysis, validation, supervision, writing–review and editing. **Miia Kivipelto:** writing–review and editing, supervision, conceptualization, funding acquisition, validation. **Anne‐Marie Boström:** writing–review and editing, supervision, conceptualization. **Berit Seiger Cronfalk:** writing–review and editing, supervision, conceptualization, formal analysis, validation.

## Ethics Statement

The study was approved by the Ethics Review Board in Stockholm, Sweden (Dnr: 2017/2400‐31/1) and conducted in compliance with *The Declaration of Helsinki*. Participants provided written informed consent once, before the start of this longitudinal study, after being informed that the study would involve interviews at multiple time points. At the start of the interview reported in this paper, the first author again informed the participant that participation in the interview was voluntary and confirmed that the participant agreed to participate.

## Conflicts of Interest

The authors declare no conflicts of interest.

## Data Availability

Research data are not shared due to privacy reasons.
